# Observation of Cation Chromophore Photoisomerization of a Fluorescent Protein Using Millisecond Synchrotron Serial Crystallography and Infrared Vibrational and Visible Spectroscopy

**DOI:** 10.1021/acs.jpcb.2c06780

**Published:** 2022-11-03

**Authors:** James M. Baxter, Christopher D. M. Hutchison, Karim Maghlaoui, Violeta Cordon-Preciado, R. Marc L. Morgan, Pierre Aller, Agata Butryn, Danny Axford, Sam Horrell, Robin L. Owen, Selina L. S. Storm, Nicholas E. Devenish, Jasper J. van Thor

**Affiliations:** † Department of Life Sciences, Imperial College London, LondonSW7 2AZ, United Kingdom; ‡ Research Complex at Harwell, Rutherford Appleton Laboratory, DidcotOX11 0FAUnited Kingdom; § Diamond Light Source, Harwell Science and Innovation Campus, DidcotOX11 0DE, United Kingdom

## Abstract

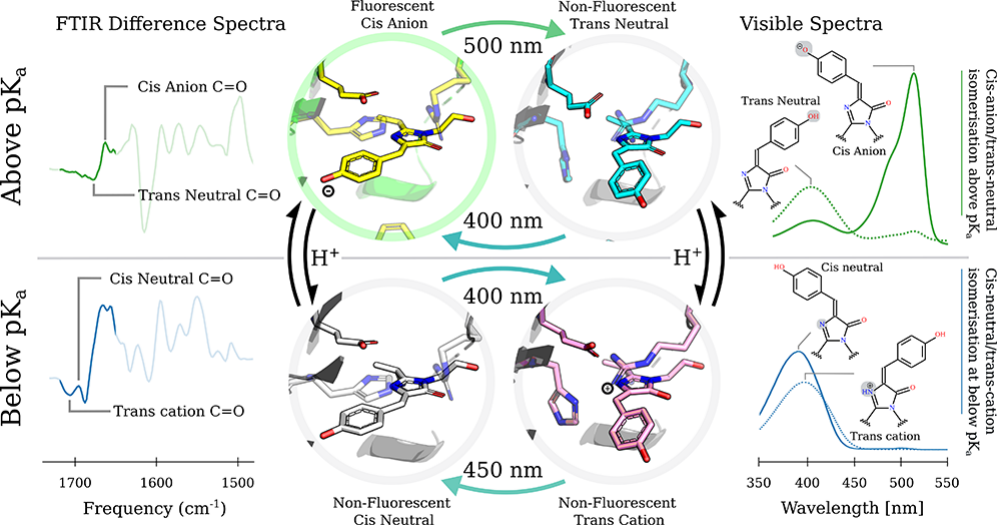

The chromophores of reversibly switchable fluorescent proteins (rsFPs) undergo photoisomerization of both the trans and cis forms. Concurrent with cis/trans photoisomerisation, rsFPs typically become protonated on the phenolic oxygen resulting in a blue shift of the absorption. A synthetic rsFP referred to as rsEospa, derived from EosFP family, displays the same spectroscopic behavior as the GFP-like rsFP Dronpa at pH 8.4 and involves the photoconversion between nonfluorescent neutral and fluorescent anionic chromophore states. Millisecond time-resolved synchrotron serial crystallography of rsEospa at pH 8.4 shows that photoisomerization is accompanied by rearrangements of the same three residues as seen in Dronpa. However, at pH 5.5 we observe that the OFF state is identified as the cationic chromophore with additional protonation of the imidazolinone nitrogen which is concurrent with a newly formed hydrogen bond with the Glu212 carboxylate side chain. FTIR spectroscopy resolves the characteristic up-shifted carbonyl stretching frequency at 1713 cm^–1^ for the cationic species. Electronic spectroscopy furthermore distinguishes the cationic absorption band at 397 nm from the neutral species at pH 8.4 seen at 387 nm. The observation of photoisomerization of the cationic chromophore state demonstrates the conical intersection for the electronic configuration, where previously fluorescence was proposed to be the main decay route for states containing imidazolinone nitrogen protonation. We present the full time-resolved room-temperature X-ray crystallographic, FTIR, and UV/vis assignment and photoconversion modeling of rsEospa.

## Introduction

A wide array of reversibly photoswitchable fluorescent proteins (rsFPs) have been developed from different fluorescent protein parent sequences and mutational studies. Crystallography and spectroscopy have shown a large variety of structural and spectroscopic behaviors.^1,2^ The first example of development of an rsFP is the GFP-like rsFP, Dronpa, which undergoes a typical reversible photoswitching behavior that involves both photoisomerization as well as protonation change of the phenolic oxygen.^3,4^ Dronpa is an example of a “negative-mode rsFP”, where the resting ground state (called the ON state) has the anionic-cis 4′-hydroxybenzylidene-2,3-dimethyl-imidazolinone (HBDI) chromophore that is highly fluorescent upon excitation with green light and has a low quantum yield of photoconversion.^5^ The metastable OFF state is the neutral-trans chromophore which has a low fluorescence quantum yield and high photoisomerization quantum yield^6,7^ that regenerates the resting ON state. Generally, the ON and OFF fluorescence states across all GFP-like rsFPs correspond to an anionic-cis chromophore and neutral-trans chromophore with only a few exceptions in the eqFP578,^4^ eqFP611^8^ and Formosa^9^ lineage which are mostly red-shifted FPs have a strongly fluorescent trans state chromophore but unclear protonation states.

Fluorescence in GFP-like rsFPs is thought to originate from the reduction of flexibility around the chromophore methyl bridge, caused by interactions with the protein chromophore pocket.^10−13^ Theoretical studies have made quantum chemistry calculations of the excited-state potential energy surfaces of the chromophore as summarized by Meech.^14^ The key findings are that excitation of the chromophore decreases the bond order over the methyl bridge. This allows an almost barrierless rotation of the double bond between the phenol ring and imidazolinone heterocyclic. This twisting motion increases the ground state energy until the S_1_ and S_0_ surfaces cross through a conical intersection at an angle around 90°.^10,11,15^ Therefore, experimental observations in solution at room temperature show almost no fluorescence: The HBDI molecule is free to twist and relax down to a “hot” ground state in a radiationless transition. Fluorescence is only observed in solution with cooling below the glass transition temperature^16,17^ or when increased friction is introduced with viscous solvents.^12^ Svendsen et al.^18^ showed experimentally that in the gas phase, at low temperatures (100 K) the anionic-cis HBDI is fluorescent as the excited state population is trapped by a barrier which reduces the rate of internal conversion through the conical intersection.^19^ This barrier effectively stabilizes the S_1_ state and reduces the rate of internal conversion which leads to increased fluorescence rates. These theoretical and experimental studies suggest that fluorescence rates will be highest when HBDI is rigidly confined to planar conformations. In proteins, the HBDI chromophore is stabilized and constrained by hydrogen bonds from surrounding residues within the chromophore environment. A study of HBDI in multiple FPs (Dronpa, rsFastLime, asFP-A143S, mTFP0.7, and IrisFP) has correlated increased planarity of the cis chromophore with the higher fluorescent yields, in agreement with experiments on isolated HBDI.^20^ Furthermore, the increased rigidity of the protein matrix surrounding the chromophore has also been associated with greater fluorescence yields, presumably due to reduced ability for twisting-related relaxation processes.^14,21,22^ It is therefore reasonable to assume that characteristics of the isolated HBDI can be used as a basis to explain the photophysical behavior in rsFPs.

The protonation of the chromophore of rsFPs has been strongly argued to modify the photochemical behavior.^10^ Olsen et al.^23^ made a computational study of the isolated HBI and found that the protonation at the phenolic oxygen determines whether a hula-twist or one-bond flip photoisomerization pathway is taken in the isomerization. Schafer et al.^24^ studied the rsFP asFP595 and argued that protonation of the imidazolinone nitrogen, in the zwitterionic state, results in dominant fluorescence decay. Similarly, Grigorenko et al.^25^ proposed the possible cationic state in a mutant of asFP595 from computational studies and also proposed the fluorescence decay to be dominant for this state. The p*K*_a_ for protonation of the isolated HBDI chromophore is 2.5 for the cationic species and 8.5 for deprotonation of the phenolic oxygen, generating the anionic state.^26^ So far, no experimental evidence has been presented for the existence of a cationic chromophore state in a fluorescent protein.

## Results

We show the first evidence of a pH-modulated switchable neutral/cationic or anionic/neutral cis/trans chromophore isomerization in a fluorescent protein at low pH (summarized in Figure 2f). Our assignment is based on (1) the red shift of the chromophore absorption band at low pH and the change in thermal recovery kinetics, (2) the emergence of a high frequency stretch in FTIR difference spectra which can only be attributed to a change in protonation state of the chromophore and (3) structural rearrangements of the dark and illuminated states on ms times-scales observed with synchrotron serial crystallography.

### UV/Vis Spectra

**Figure 1:**
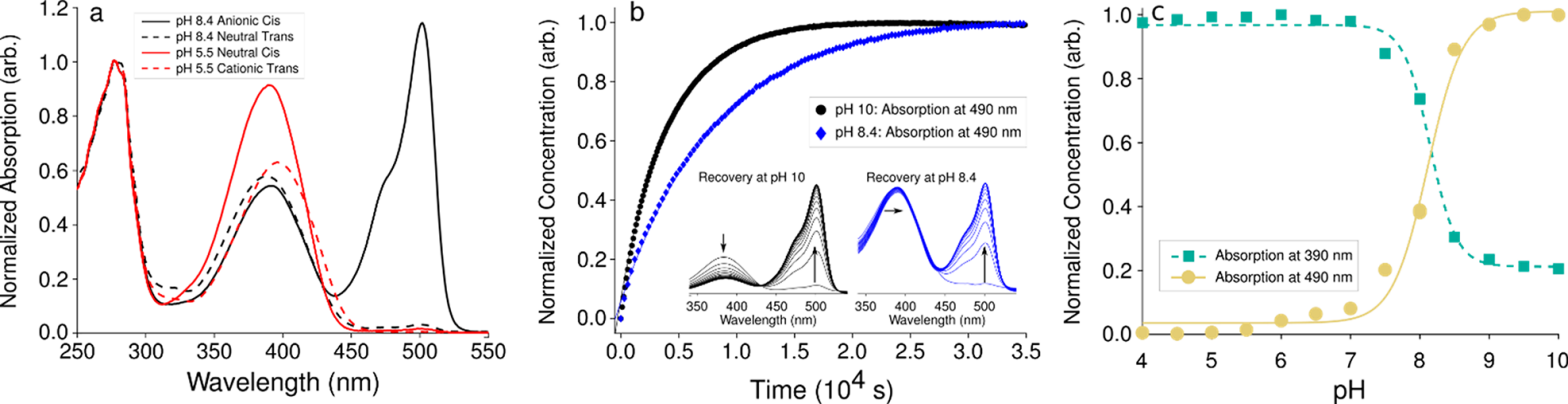
(a) Normalized UV/vis absorption spectra of rsEospa in the cis and trans states at acidic and neutral pH. At pH 5.5 the trans state was formed after illumination with 405 nm light, and at pH 8.4 the trans state was formed after illumination with 488 nm light. The anionic, neutral, and cationic assignments are discussed in the main text. The spectra are normalized at 280 nm. (b) Thermal recovery rates of the anionic-cis peak at 490 nm at pH 8.4 and 10 after full conversion with 488 nm light. (c) Absorption of the 490 and 390 nm peaks of unilluminated rsEospa as a function of pH. The solid lines follow the Henderson–Hasselbalch equation to fit the p*K*_a_ of 8.1.

The UV/vis absorption spectra of rsEospa dramatically shift as a function of pH (Figure 1a). We observe a 503 nm absorption maximum at pH 10 and pH 8.4 which is assigned to the anionic-cis chromophore state as in Dronpa.^18^ With decreasing pH, we observe a bleach at 503 nm correlated with an increase in absorption at 390 nm (Figure 1c). This spectra shift is interpreted as protonation of the chromophore to a neutral state (confirmed as cis chromophore by crystallography below) for which we fit a p*K*_a_ of 8.1. Illumination with 488 nm light at pH 10 generates the 392 nm absorption peak, assigned to a neutral-trans state which recovers thermally back to the anionic-cis state at room temperature following first-order kinetics (Figure 1b).

At pH 8.4, strong absorption is still seen at 503 nm, but a new absorption feature at 392 nm is also present. We propose assignment of a minor neutral-cis chromophore on the basis of the absorption shift upon illumination. Illumination with 488 nm causes a small increase in the intensity of the 392 nm peak and a blue shift of 5 to 387 nm. The recovery of this state follows a two-step kinetic scheme (Figure 1b and Supplementary Results). At pH 5.5, the dark state shows an absorption peak only at 389 nm which is assigned to a neutral-cis state. This absorption profile is similar to the trans state at pH 8.4 but stronger. Excitation of the neutral-cis with 400 nm light causes a bleach in the 389 nm peak and a + 7 nm redshift assigned to the formation of a cationic-trans chromophore. This assignment is supported by studies of the electronic spectra of HBDI where peaks at 385, 395, and 450 nm are known to correspond to the anionic, cationic and neutral chromophore,^3,14^ although definitive evidence is provided in the FTIR and crystallography experiments described below. Excitation at 430–450 nm of the cation-trans state then reversibly switches back to the original “dark” state spectra, the neutral-cis (Figure S10). The fluorescence emission for both the neutral-cis and trans states was weak, while the anionic-cis state showed appreciable fluorescence with excitation between 450 and 500 nm. The fluorescence spectra exhibit a Stokes shift of 9 nm.

### FTIR Difference Spectra Assignment

**Figure 2:**
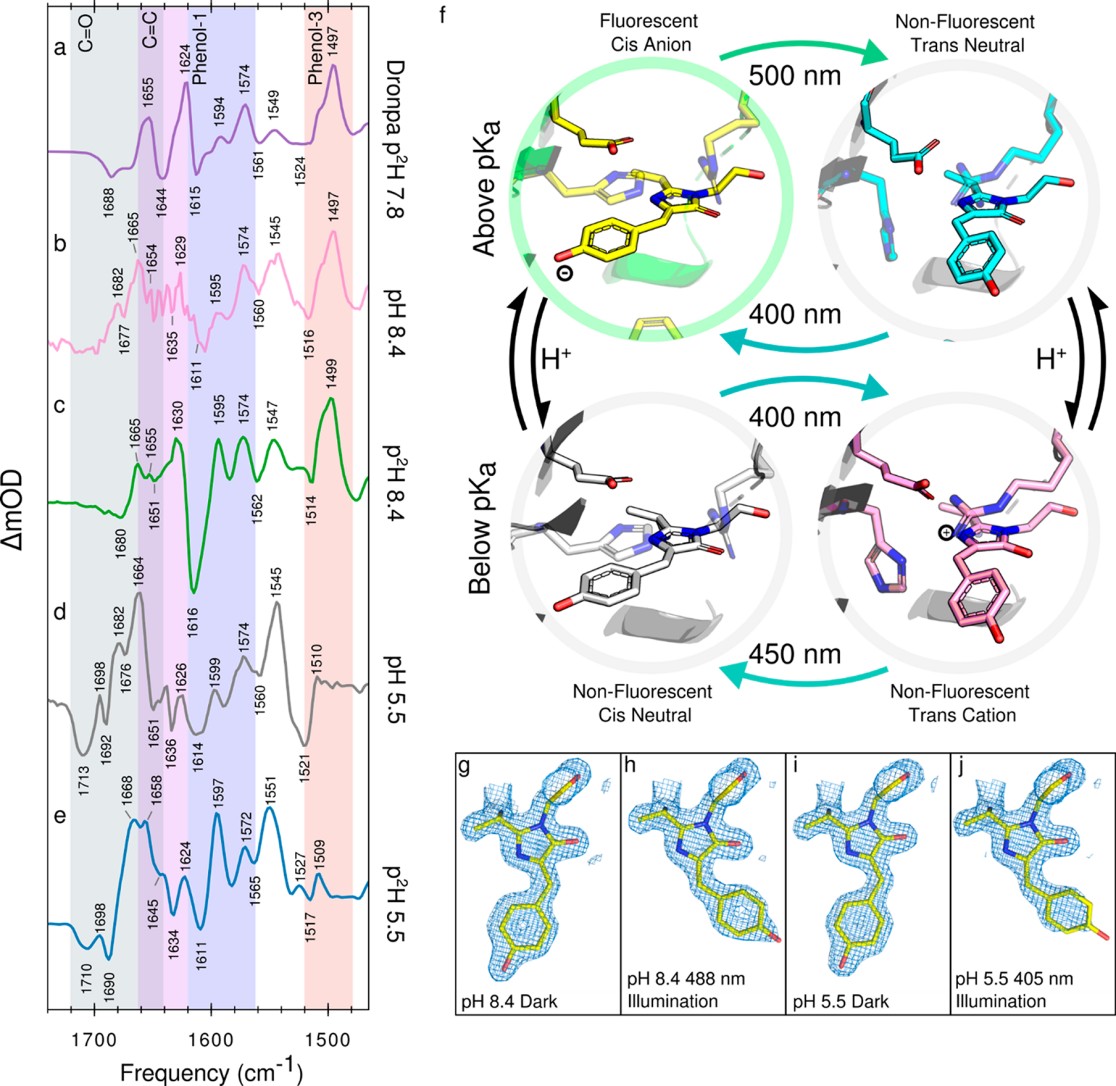
(a–e) FTIR difference spectra for rsEospa and the wild-type Dronpa relative to the OFF state (dark-illuminated spectra). At pH/p^2^H 8.4 the sample was illuminated with 488 nm light; at pH/p^2^H 5.5 the sample was illuminated with 405 nm light. (f) Proposed schematic for switching above and below the p*K*_a_. (g–j) 2F_o_–F_c_ chromophore omit–density maps of the serial synchrotron crystallography structures for conditions: unilluminated pH 8.4, 488 nm illuminated at pH 8.4, unilluminated at pH 5.5, and 405 nm illuminated at pH 5.5.

FTIR difference spectra of rsEospa were recorded for H_2_O and ^2^H_2_O conditions at pH/p^2^H 5.5 and pH/p^2^H 8.4 (Figure 2b–e and Experimental Methods). A majority of the spectral features observed in Dronpa^6,27−29^ (Figure 2a) are also present in rsEospa, so initial mode assignment at neutral pH follows those reported by Warren et al.^6^ We identify signals in the difference spectrum belonging to the chromophore, Arg66, and the protein backbone. The C=O stretching mode of HBDI is known to upshift with decreased electron density of the imidazoline ring which therefore acts as a direct reporter for the chromophore protonation state.^30^ We focus our discussion in the main text to this stretching mode and report the structural interpretation of the lower frequency modes in the supplementary results. At pH/p^2^H 8.4 the C=O modes at 1665/1655 cm^–1^ and 1677/1680 cm^–1^ for the dark and illuminated states are assigned to anionic-cis and neutral-trans chromophores, respectively, based on the calculated frequency upshift and ^1^H/^2^H sensitivity of a hydrogen-bonded HBDI.^6^ An assignment to a cis-zwitterionic or trans-cation would be unprecedented as both the visible spectra and FTIR difference spectra closely resemble Dronpa.

Under the dark condition at pH/p^2^H 5.5, we observe a high frequency mode at 1698/1698 cm^–1^ which is assigned to the C=O mode of a *neutral*-cis chromophore. Harmonic frequency calculations suggest the neutral-cis will show weakened absorption for the phenol-3 band (1/160×), stronger absorption and upshift of the C=C band (6× and +30 cm^–1^) and an upshifted C=O stretch (+70 cm^–1^) compared to the anionic-cis chromophore.^6^ At pH/p^2^H 5.5 we observe shifts in intensity and frequency that agree with these predictions (Table 1). Combined with the large blueshift in electronic spectrum (Figure 1a), this is strong evidence for the neutral protonation state of the cis chromophore at pH 5.5 as opposed to the anionic-cis at pH 8.4.

**Table 1:** Photoconverted State Assignments of rsEospa at p^2^H/pH 5.5 and 8.4^a^

	dominant component	p^2^H 8.4	pH 8.4	p^2^H 5.5	pH 5.5
dark state	C=O	1655	1665	1698	1698
	Arg66_asym_(CN_3_H_5_^+^)	1665	1682	1668	1682
	C=C	1630	1629	1664	1658
	Arg66_sym_(CN_3_H_5_^+^)	1595	1595	1597	1599
	Phenol-1	1574	1574	1572	1574
	C=N/C=C	1547	1545	1551	1545
	Phenol-3	1499	1497	1509	1510
	^13^C_5_ sensitive	N.D.	1348	N.D.	1346
	Phenol	1150	1148	1155	1153
					
illuminated state	C=O	1680	1677	1710/1690	1713/1692
	C=C	1651	1651	1645	1651
	Phenol-1	1616	1611	1611	1614
	C=N/C=C	1562	1560	1565	1560
	phenol-3	1514	1516	1517	1521
	Phenol	1177	1176	1176	1177

a All values in cm^–1^.

**Figure 3:**
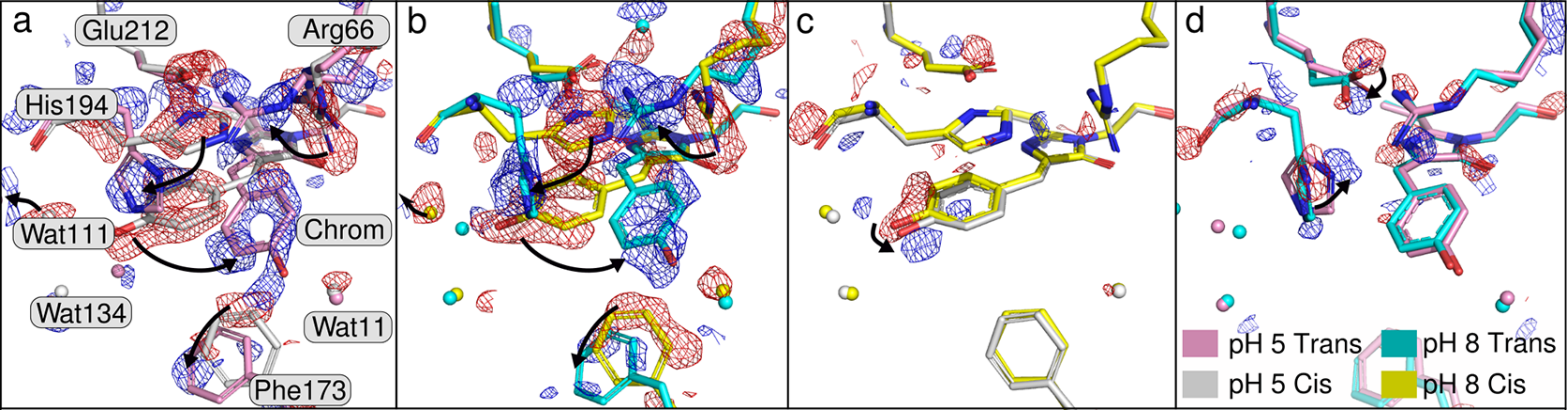
Q-weighted difference electron density maps of different states of rsEospa plotted in red and blue at 3σ level. Shown in purple, gray, cyan, and yellow are the refined coordinates for pH 5.5 trans, pH 5.5 cis, pH 8.4 trans, and pH 8.4 cis, respectively. Difference maps are plotted for (a) pH 5.5 405 nm–pH 5.5 dark, (b) pH 8.4 488 nm–pH 8.4 dark, (c) pH 5.5 dark–pH 8.4 dark, and (d) pH 5.5 405 nm–pH 8.4 488 nm.

Under the illuminated condition, we observe difference spectra peaks at 1713 and 1692 cm^–1^ at pH 5.5 and at 1710 and 1690 cm^–1^ at p^2^H 5.5 which we assign to the C=O stretch of the cationic-trans chromophore. The 1713/1710 cm^–1^ (^1^H/^2^H) frequency is in a highly characteristic spectral region for FTIR spectroscopy of proteins.^31^ A COOH carboxylate assignment is usual for the frequency but the small 3 cm^–1^ downshift with ^1^H/^2^H exchange cannot support an assignment. Furthermore, an additional strong asymmetric ν_asym_(COO^–^) mode from a carboxylate deprotonation would also be expected close to 1567 cm^–1^ in ^2^H_2_O and between 1556 and 1560 cm^–1^ for ^1^H_2_O^31^ which is not observed (Figure 2d,e). In addition, due to a putative deprotonation of a carboxylate in the illuminated state, an additional upshifted positive Glu(COOH) stretch would be expected for the dark state which is not observed (Figure S6). The crystallographic results indicate electron density differences on Glu212 (Figure 3d). Therefore, the FTIR spectroscopy indicates that there are no (de)protonation reactions either at pH 5.5 or 8.4. Instead, the 3 cm^–1^ downshift of 1713/1710 cm^–1^ C=O mode with ^1^H/^2^H exchange must arise from mode mixing of an otherwise unionizable chromophore C=O group. The extensive FTIR, TR-IR, and Raman literature shows that either neutral or anionic HBDI chromophores in FPs have a upper limit of 1695 cm^–1^ for the imidazolinone C=O stretching mode.^32−37^ Possible mechanism other than ionization for strongly upshifting this mode are increased localization, significant reduction of the dielectric constant of the medium, or changes in geometry^7^ which are not seen by crystallography. The imidazolinone ring does not allow change of localization as the ring deformation is included in the C=O stretching mode displacement. The assignment of the 1713/1710 cm^–1^ frequency to the cationic C=O stretching mode is therefore confidently made.

**Figure 4:**
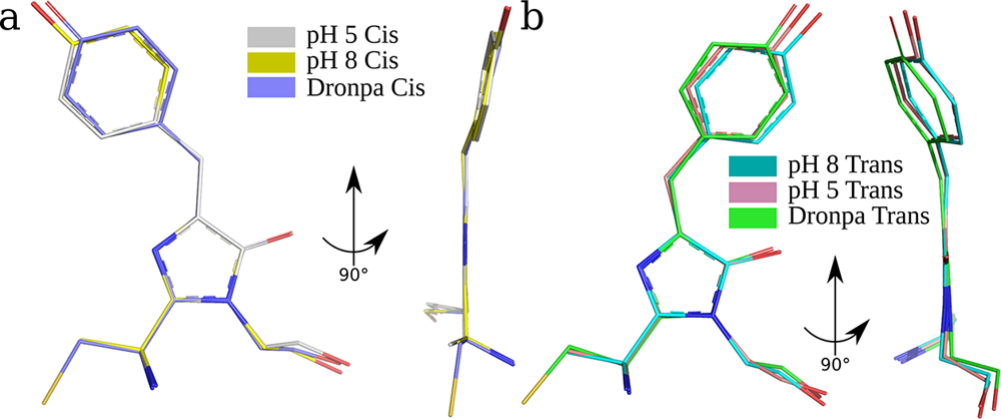
Chromophore alignment from the crystal structures of rsEospa at pH 5.5, pH 8.4 and Dronpa. The Dronpa structures were obtained from the Protein Data Bank (IDs: 2POX (trans) and 2IOV (cis)).

Furthermore, the frequency position indicates that the cationic C=O group is strongly hydrogen-bonded. Harmonic frequency calculations of HBDI at the DFT B3LYP/6-311+g(d,p) level in vacuum predict an upshift in ν(C=O) of +54 cm^–1^ between neutral and cationic trans-state HBDI.^6^ This is experimentally confirmed in the FTIR and Raman measurement of HBDI in neutral and cationic form which found state C=O frequencies at 1699 and 1749 cm^–1^, respectively.^37^ Including the hydrogen bonding by addition of a water to cationic HBDI downshifts the ν(C=O) by 13 cm^–1^. The experimental observation of the downshifted 1713 cm^–1^ frequency thus indicates the new formation of a strongly hydrogen bonded environment upon chromophore ionization. In this study, a shift of +10 cm^–1^ is observed for the ν(C=O) mode between the neutral state at p^2^H 8.5 and the cationic state at p^2^H 5.5 (Figure 2c,e). Small frequency shifts are expected if the chromophore twisting angles differ significantly;^7^ however, the TR-SSX structures show the trans conformations are very similar (Figure 4 and Table S4) meaning any large shifts in chromophore stretching frequencies are unlikely to be caused by changes to the chromophore geometry. Furthermore, harmonic frequency calculations^6^ suggest the cationic-trans should have downshifted ν(C=C), Phenol-1, Phenol-2, ν(C=N/C=C), and Phenol-3 modes. The assignments to these modes all follow this trend except the Phenol-3 stretch which is upshifted compared to the neutral-trans assignment (supplementary results). We can therefore confidently conclude that the shift of 10 cm^–1^ in ν(C=O) between p^2^H 8.4 to 5.5 is due to the formation of a cationic trans-state at low pH.

Finally, the protonation state of Glu212 (equivalently Glu211/Glu222 in Dronpa/GFP) is predicted to be neutral at pH 8 as it is involved in the regulation of the proton transfer mechanism.^38,39^ In avGFP, Glu222 maintains an H bond through serine 65 which stabilizes the anionic state of the glutamic acid, which acts as a proton acceptor from the photoacidic chromophore. In Dronpa and GFP S65T, there is no donating hydrogen bond to Glu211/222, so the neutral state is maintained.^40^ Since rsEospa is structurally most similar to Dronpa and there is no stabilizing H bond formed with Glu212, a neutral Glu212 is most likely maintained at pH 8.4. If the Glu212 is neutral at pH 8.4, then it will not undergo deprotonation at pH 5.5. Therefore, the 1710 cm^–1^ band must originate from the chromophore and is assigned to an additional C=O stretch.

### Millisecond Time-Resolved Serial Synchrotron X-ray Crystallography

Time-resolved serial crystallography structures at room temperatures were collected using a 1 ms optical-pump X-ray-probe delay. In total, 61,864 crystal diffraction images were merged into 6 data sets (Experimental Methods) for the following conditions (with the number of merged diffraction patterns in parentheses): pH 8.4 under dark conditions (10,790), pH 8.4 and 488 nm illumination (9,342), pH 8.4 and 405 nm illumination (11,917), pH 8.4 and 488 nm illumination followed by 405 nm illumination (preconversion followed by a flash) (7,696), pH 5.5 under dark conditions (9,015), and pH 5.5 and 405 nm illumination (13,104). The limiting resolution was found to be 1.75 Å as determined by a value of 0.5 in Fourier shell correlation^41^ (Tables S1 and S2). The refined unit-cell size varied less than 0.9% in volume for each condition, allowing the calculation of isomorphous Fourier difference electron density maps using a single set of “dark” phases. Refinement gave coordinates with *R*_Work_/*R*_Free_ values of 0.19–0.20/0.22–0.23. Merging statistics, such as CC_1/2_ and *R*_Split_, were all appropriate for TR-SSX data and maintained similar values for all conditions.

At pH 8.4, the 2F_o_–F_c_ chromophore omit–density (Figure 2g,h) confirm this cis (trans) conformation for the unilluminated (488 nm illuminated) structures. Illumination of the pH 8.4 trans state with 405 nm light (after 488 nm preconversion) is shown to regenerate the cis state, as is typical in negatively switching FPs (supplementary results). At pH 5.5 we see a striking difference in the photoswitching behavior. The unilluminated structure shows the cis chromophore, while illumination with 405 nm light accumulates the trans state with high yields. To estimate the efficiency of switching we refine the chromophore cis/trans occupancy (Table S3 and Figure S4). Here, the highest level of structural homogeneity is seen in the pH 5.5 dark state at 96/73 (4/27)% cis (trans) occupancy for the *R*/*R*_Free_, respectively. Population transfers calculated from the occupancy refinement suggest the low pH cis–trans isomerization reaction occurs with higher yields than the neutral pH trans–cis. As judged from the *R*-factor minima, population transfers of 30 and 17% for these reactions at pH 5.5 and 8.4, respectively.

To directly compare changes in structure due to pH and illumination conditions we calculate the *Q*-weighted difference electron density (DED) (Figure 3).^42,43^ A majority of DED features are localized to the chromophore and surrounding residues although we also observe a shift in the crystal contacts at low pH (supplementary results).

In the pH 8.4 488 nm–pH 8.4 dark and pH 5 405 nm–pH 5 dark DED (Figure 3a,b), we observe significant signal indicating the movement of the chromophore, Arg66, Phe173, His194, and Glu212. The overall similarity of these movements suggest the light-induced conformational changes of the protein at neutral and low pH are similar despite such different spectral characteristics. Comparing the DED of the cis pH 5.5 and cis pH 8.4 structures (Figure 3c), we observe a flattening of the chromophore phenol ring and a shift in coordinating water molecules around the chromophore at low pH. This shift disrupts a donating hydrogen-bond network through Thr159, Water134, Water111, and Water128 to the chromophore phenol group (Figure S3a,c). This is further evidence for the protonation of the anionic-cis chromophore at low pH, as, presumably, the neutral chromophore phenol cannot support the extra hydrogen bond, unlike the anion.

The DED of the trans structures at pH 8.4 and 5.5 (Figure 3d) indicate movement of His194 and Glu212 and show significant negative density close to the N4 and C=O of the imidazolinone ring. The Glu212 carboxyl oxygen to imidazolinone-ring nitrogen (N4) distance is reduced from 3.76 Å at pH 8.4 to 2.54 Å at pH 5.5. We interpret this rearrangement as the formation of a H-bond which is made favorable by protonation of the chromophore. We note that the low pH Glu212 exhibits higher atomic *b*-factors compared to the neutral pH structure (Figure S8); however, we do not believe this increase accounts for the magnitude of the displacement observed. We therefore conclude that the majority of trans chromophore structural changes between pH 8.4 and 5.5 are due to the formation a cationic-trans chromophore compared to the neutral.

On their own, these structural changes observed in the crystallography are not definitive for assigning the protonation state of the chromophore as many conformations of hydrogen bonding networks have been observed in GFP like proteins;^39,44,45^ however, our observations provide direct support for UV/vis and FTIR assignments.

## Discussion

RsEospa shows cis–trans isomerization at both acidic and neutral pH with similar atomic coordinate modification but distinctively different visible excitation requirements and spectral features. At acidic pH, this isomerization occurs reversibly with a change in protonation state to a trans-cation chromophore state, a new class of reaction not previously observed in rsFPs. In contrast to previous suggestions the cation state is not fluorescent and instead is reactive to support photoisomerisation.^23,24^ At neutral pH, the isomerization occurs from an anionic-cis to a neutral-trans which is also reversible. The ultrafast switching mechanism of negative switching FPs in the Anthozoa family is debated and early steps of the isomerization reaction remain unclear from current ultrafast spectroscopy measurements, especially in the forward direction (cis–trans).^6,7,29,46^ The high yields of the cis–trans reaction at pH 5.5 relative to the neutral pH trans–cis reaction are consistent with the quenching of fluorescence. These characteristics make rsEospa a unique target for gaining insight into excited state motions of the cis–trans reaction which is poorly studied in negative-type switching FPs.

Both the shifts in visible spectra and the thermal recovery rates modeling suggest an acid base equilibrium of the chromophore cis state as also previously suggested in Dronpa.^47^ While the protonation state changes are confirmed by FTIR, TR-SSX confirms the isomerization state and shows the reaction is complete after 10 ms. This is expected as the slowest rate constants in similar rsFPs, rsEGFP2, Dronpa, and IrisFP, are assigned to the deprotonation step which is on microsecond time scales.^6,29,46,48^ Confirmation of the visible spectral assignments is seen in the crystal structures. The blue-shifted 380 nm visible absorption is clearly due to a cis conformational as seen in the pH 5.5 dark coordinates, while the 390 nm peak, caused by 405 nm illumination of the pH 5.5 species, is clearly due to the trans state. The pH 5.5 trans peak is red-shifted compared to the pH 8.4 one. This is most likely due to a combination of the change in protonation state of the trans conformer and stabilization caused by the hydrogen bond formed with Glu212 (Figure S3) which is not seen at pH 8.4.

The identification of the ν(C=O) mode at the characteristic frequency of 1713 cm^–1^ is unusual for FTIR spectroscopy of protein samples when the lack of ^1^H/^2^H downshift excludes assignment to carboxylate COOH stretching. There is one notable other example in the literature, which is the cyanobacterial phytochrome Cph1^49,50^ and the plant phytochrome A from *Avena sativa*.^51^ FTIR experiments used isotope labeling of the tetrapyrrole chromophores to identify the ν(C=O) modes in the 1700–1750 cm^–1^ region. It was concluded that the upshifted ν(C=O) frequencies were the result of twisted chromophore conformations.

The protonation states of the pH 8.4 and 5.5 dark conditions are confirmed by the FTIR difference spectra which support an anionic-cis and neutral-cis states, respectively. The anionic-cis state assignment is supported by similar visible and IR spectral features in the FPs of Dronpa and GFP.^6,7,30,32^ The neutral-cis assignment is supported by the large blueshift in electronic spectra and agreement of harmonic frequency calculations^6^ with the FTIR difference spectra. Calculations of the HBDI neutral-cis chromophore^6^ suggest weakened absorption at the phenol-3 cis peak, a stronger C=C band, and an upshifted C=O stretch which is seen in the pH/pD 5.5 dark 405 nm difference spectra. Together with the 389 nm absorption band position, the FTIR spectrum provides strong evidence for the neutral protonation state of the cis chromophore at pH 5.5 as opposed to the anionic-cis at pH 8.4. Furthermore, a change in hydrogen bonding is seen around the chromophore in the low pH cis crystal structure. A donating network through Thr159 is disrupted presumably because the neutral chromophore phenol cannot support the extra hydrogen bond, unlike the anion. A cis-zwitterionic state, with protonation of the imidazolinone nitrogen, is calculated to show a strong Phenol-2 absorption as an on-state peak.^6^ This is not observed in the difference spectra meaning the assignment of a zwitterionic state would be unjustified. The assignment of the nonfluorescent trans state at pH 5.5 to the cationic chromophore is made with confidence on the basis of the frequency position of the ν(C=O) mode. It is further strongly supported by the UV/vis measurements. The cationic nature and its hydrogen bonding is illustrated by harmonic frequency calculations.^6^ Calculations predict an upshift in ν(C=O) of +54 cm^–1^ between neutral and cationic-trans state HBDI molecule. In this study, a shift of +10 cm^–1^ is observed between the neutral and low pD measurements. Some shifts are expected if the chromophore twisting angles differ significantly;^7^ however, the TR-SSX structures show the trans conformations are very similar (Figure 4 and Table S4) meaning any large shifts in chromophore stretching frequencies are unlikely to be caused by changes to the chromophore geometry. We can therefore confidently conclude that considering the restrained and slightly distorted trans geometry of the chromophore (compared to harmonic calculations),^6^ the shift of 10 cm^–1^ in ν(C=O) between pD 8.4 to 5.5 is due to the formation of a cationic trans-state at low pH. Furthermore, harmonic frequency calculations suggest the cationic-trans should have downshifted ν(C=C), Phenol-1, Phenol-2, ν(C=N/C=C), and Phenol-3 modes. The assignments to these modes all follow this trend except the Phenol-3 stretch which is unexpectedly upshifted compared to the neutral-trans assignment.

## Conclusions

We have shown using FTIR and visible spectroscopy as well as ms room temperature X-ray crystallography the unusual photo switching behavior of the rsFP rsEospa. This behavior is remarkable in two respects. First, the FTIR spectroscopy definitively supports the cationic chromophore state as the OFF state which is not fluorescent, as previously suggested,^12,15,16^ but can efficiently support photoisomerization. The assignment is furthermore strongly supported by the formation of a hydrogen bond of the imidazolinone nitrogen with the Glu221 carboxylate, which does not occur in Dronpa. Second, we note the efficient cis–trans isomerization at pH 5.5. Current time-resolved studies reported are for the trans–cis direction on accord of the generally high quantum yield of photoisomerization. Specifically, at pH 5.5, the cis–trans photoisomerization of rsEospa presents an opportunity to study this reaction using ultrafast methods.

## Supporting Information Available

Detailed methodology, discussion of the vibrational mode assignment, crystallography data tables and supplementary figures (PDF)
