# Supplementary material for: Observation of Cation Chromophore Photoisomerization of a Fluorescent Protein Using Millisecond Synchrotron Serial Crystallography and Infrared Vibrational and Visible Spectroscopy
Source: J Phys Chem B. 2022 Nov 3;126(45):9288–96. doi: 10.1021/acs.jpcb.2c06780 (PMC9677427; doi:10.1021/acs.jpcb.2c06780)
Supplement: Supplementary file 1 — jp2c06780_si_001.pdf [file jp2c06780_si_001.pdf]

Supplementary Material: Observation of Cation Chromophore Photoisomerization of a Fluorescent Protein Using Millisecond Synchrotron Serial Crystallography and Infrared Vibrational and Visible Spectroscopy

James M. Baxter[1], Christopher D.M. Hutchison[1], Karim Maghlaoui[1], Violeta Cordon-Preciado[1], R. Marc L. Morgan[1], Pierre Aller[2,3], Agata Butryn[2,3], Danny Axford[3], Sam Horrell[3], Robin L. Owen[3], Selina L. S. Storm[3], Nicholas E. Devenish[3], Jasper van Thor*[1].

[1]Department of Life Sciences, Imperial College London, London, SW7 2AZ, UK,

[2]Diamond Light Source, Harwell Science and Innovation Campus, Didcot, UK;

[3]Research Complex at Harwell, Rutherford Appleton Laboratory, Didcot, UK

Experimental Methods

UV/Vis Steady State Spectra & Thermal Recovery

The sample is a recombinantly expressed triple mutant L62A S142A M159T of the rsFP Skylan-NS [1] which in-turn is based on EosFP sequence [2]. The protein was expressed in *E.coli*, purified with Ni-NTA and size exclusion chromatography and used for all spectroscopy and crystallography experiments. UV/Vis spectra acquired from 200-1000 nm of the RsEospa protein at around 2 mg/ml concentration at pH 5.5, pH 8.4 and pH 10 in a low salt buffer (50 mM buffering acid, 100 mM NaCl) buffered with 50 mM MES, Tris and HEPES respectively. Samples were converted using either a 405 or 488 nm laser. Thermal recovery measurements were made by pre-converting the sample with a 488 nm laser then measuring spectra every 30 seconds using a 0.5 s exposure time over 8 hours. The exposure time and frequency of measurement were kept to a minimum to reduce the actinic behavior of the white light probe. Data were fitted and analyzed using custom-written MATLAB scripts.

FTIR Difference Spectra

FTIR samples in H_2O conditions were prepared by concentrating the protein to approximately 95 mg/ml in 50 mM Tris-HCl and 100 mM NaCl at pH 8.4 (or 50 mM MES-HCl and 100 mM NaCl at pH 5.5). 15 µl of concentrated protein sample was then placed in a Harrick cell between two CaF_2 UV polished-windows (1 mm and 2 mm) using a 6 µm Teflon spacer, giving a sample OD of 0.7 at 490 nm. To prepare deuterated samples, the same buffers were prepared using ^2H_2O at either p^2H 8.4 or p^2H 5.5. and water content was minimized below 0.2%. The final sample concentration in ^2H_2O conditions was 60 mg/ml and was placed in a Harrick cell between two CaF_2 UV polished-windows (1 mm and 2 mm) using a 25 µm Teflon spacer, giving a sample OD of 0.8 at 490 nm.

Spectra were acquired using a Biorad ISF 66/S at 4 cm^{-1} resolution using a liquid nitrogen-cooled MCT-A detector. CW laser illumination with wavelengths of 405nm (500 mW) and 488 nm (100 mW) was directed through a side port of the spectrometer and defocused onto the Harrick cell using a misaligned 1:1 telescope. Illumination times were between 20-30 s to achieve full conversion. Dry air was used to purge the spectrometer for 1-2 hours between opening of the sample enclosure.

Time Resolved Serial Synchrotron Crystallography at Low and Neutral pH

Crystals were prepared in batch conditions at pH 8.4 with 25% PEG3350 0.2 M Lithium Sulfate, 0.1 M Tris-Cl at a final protein concentration of 15 mg ml^{-1} with a final volume of 500 µl. These conditions formed needle-shaped crystals of dimensions around 2×2×10 µm and density around 10^7-10^8crystals/ml. Crystals at pH 8.4 were diluted by a factor of 8 before being loaded onto the fixed target support. Crystals at pH 5.5 were prepared by centrifuging the slurry at 1 g for 2 minutes followed by removal of 300 µl of mother liquor which was replaced with 25 % PEG3350 0.2 M Lithium Sulfate 0.1 M MES at pH 5.5. This was repeated 3 times to ensure the pH was close to 5.5. A color change from bright to dim yellow was immediately observed on exchanging the buffer which indicated the pH was below the pK_a value. Preliminary experiments on larger batch trials showed the final pH was 5.5 ± 0.1 after buffer replacement.

Time-resolved serial synchrotron crystallography (TR-SSX) measurements were performed at the beamline I24, Diamond Light Source . An unfocused 100 mW laser of wavelength 488 nm and beam profile around 3×2 mm was directed onto a fixed target serial crystallography chip [3] shown in figure S2a. Lasers of wavelength 405 and 488 nm laser were combined collinearly using a long pass dichroic mirror (Thorlabs, FEL0450). The 405 nm beam was attenuated with a 1 OD neutral density filter (Thorlabs) and focused with a lens (300 mm focal length) placed just before the dichroic mirror.

This focused the 405 nm beam and left the 488 nm unfocused. TTL modulation was used to set a pulse length of 1 ms for the 405 nm laser. The half-life of the laser rise time was around 600 µs and approximately linear, implying that the laser reached 500 mW by the end of the pulse. The fall time was very short (around 10 µs). The 405 nm laser was focused to a spot size of 12×41 µm as measured by a knife-edge scan (figure S2b) and had a CW power of 21.0 mW at the focus. The laser and X-ray focal spots were aligned to overlap with each other with the 405nm laser illuminating a single aperture of the chip immediately prior to exposure to X-rays. The pre-illumination routine consisted of exposing an entire city block to the 488 nm laser for 10 s. The X-ray diffraction data were taken using an X-ray exposure time of 10 ms using unattenuated 0.9801 Å radiation of flux ~10^{12} photons/s beam and beam-size of 7×7 µm. To record diffraction patterns a Dectris Pilatus3 6M detector was used at a distance of 320 mm. Initially both DIALS [4] and CrystFEL[5] using indexing methods XGANDALF

[6] and iMOSFLM [7] were used for data reduction. It was found that XGANDALF gave the best merging statistics so was used for processing downstream. Detector distance was only coarsely refined in steps of 1 mm as the unit cell distributions and indexing rates did not change significantly with different detector distances. Data from CrystFEL was merged ignoring partialities.

Structure factors were calculated from intensities using Phenix with the massage option which treats negative structure factors with a scaling similar to the French & Wilson method. [8] Q-weighted difference electron density was calculated using custom written scripts based on [9] and refinement of structures was done using restrained refinement in REFMAC5 [10] and real-space refinement in coot.[11] Omit maps were calculated by setting the chromophore occupancy to 0 and per forming '0' rounds of refinement in REFMAC5. Approximate occupancies were calculated through minimization of crystallographic R-factors. A model containing both cis and trans conformations were generated and the occupancy changed in 1 % intervals from 0 % cis (100 % trans) to 100 % cis (0% trans) and R-factors calculated after 5 cycles of rigid body refinement. The minima of R_{Free} and R_{Work} were used to estimate the population of cis/trans conformation in the dataset.

Supplementary Results

Interpretation of Thermal Recovery Rates:

A scheme to account for the change in the rate of the thermal recovery is shown below. An initial state of the neutral trans conformation (A_{Trans}) which can directly isomerize (upon photo-excitation) and deprotonate with rate k_{Iso} to form the anionic cis species (B_{Cis}). An alternative pathway of isomerization to a neutral cis (A_{Cis}) with rate constant k_{AA} and acid base equilibrium with forward and back rate constants of k_{AB} and k_{BA} to regenerate the anionic cis state.

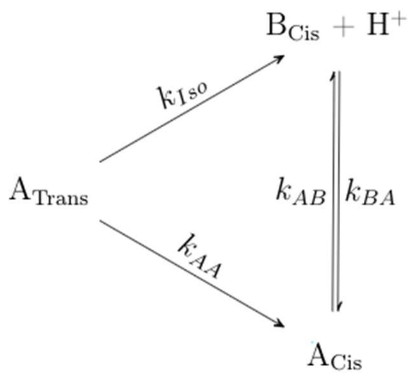

The acid base equilibrium constant, K_a can be written in terms of the concentration of the reactants (indicated with square brackets), the buffer pH and the pK_a of rsEospa which was measured in to be 8.1 (figure 1c):

$$[A_{Cis}] = \frac{[B_{Cis}][H^+]}{K_a} = \frac{[B_{Cis}]10^{-pH}}{10^{-pK_a}}$$

The rate equations for the scheme suggested above can be written as:

$$\frac{\partial[A_{Trans}]}{\partial t} = -k_{AA}[A_{Trans}] - k_{Iso}[A_{Trans}]$$

$$\frac{\partial[B_{Cis}]}{\partial t} = k_{AB}[A_{Cis}] - k_{BA}[B_{Cis}][H^+] + k_{Iso}[A_{Trans}]$$

$$\frac{\partial[A_{Cis}]}{\partial t} = -k_{AB}[A_{Cis}] + k_{BA}[B_{Cis}][H^+] + k_{AA}[A_{Trans}]$$

$$\text{Substituting: } k_1 = k_{AB}10^{-pH+pK_a} - k_{BA}10^{-pH}$$

$$\frac{\partial}{\partial t}\begin{pmatrix} A_{Trans} \\ A_{Cis} \\ B_{Cis} \end{pmatrix} = \begin{pmatrix} -k_{AA} - k_{Iso} & 0 & 0 \\ k_{AA} & 0 & -k_1 \\ k_{Iso} & 0 & k_1 \end{pmatrix} \begin{pmatrix} A_{Trans} \\ A_{Cis} \\ B_{Cis} \end{pmatrix}$$

Solving for the Eigenvalues gives:

$$\lambda_1 = 0 \text{ (Trivial)} \quad \lambda_2 = k_1 \quad \lambda_3 = -k_{AA} - k_{Iso}$$

With the corresponding Eigenvectors:

$$v_1 = \begin{pmatrix} 0 \\ 1 \\ 0 \end{pmatrix} \quad v_2 = \begin{pmatrix} 0 \\ -1 \\ 1 \end{pmatrix} \quad v_3 = \begin{pmatrix} -(k_1 + k_{AA} + k_{Iso})/k_{Iso} \\ (k_1 + k_{Iso})/k_{Iso} \\ 1 \end{pmatrix}$$

Defining $k_{Obs} = k_{AA} + k_{Iso}$ this gives a solution of the form:

$$[B_{Cis}](t) = \alpha_0 + \alpha_1 \exp(k_1 t) + \alpha_2 \exp(-k_{Obs} t)$$

Here, k_1 follows a sigmoid which becomes significant at pHs below the pK_a. This means in the high pH limit (pH 10) the rates will follow first order rate laws whilst second order rate law will be observed close to and below pH 8.17. Fitting the pH 8.4 thermal recovery (figure 1b) gives values of 0.3, 0.77, 9.2e-7 s^{-1}, 0.97, -1.2e-4 $s^{-1}$for α_0, α_1, k_1, α_2 and k_{Obs}.

The pH 10 data was fitted by using the high pH limit of:

$$[B_{Cis}](t) = \alpha_0 + \alpha_1 + \alpha_2 \exp(-k_{Obs} t) = \beta + \alpha_2 \exp(-k_{Obs} t)$$

With coefficients of 0.99, -0.93 and 1.2e-4 $s^{-1}$for β, α_2 and k_{Obs}.

Vibrational Mode Assignment

A majority of spectral features are conserved between the Dronpa and rsEospa (figure S6 a-c) although differences are seen particularly in the fingerprint region (1280 - 1380 cm^{-1}) and in the carbonyl region (between 1640-1680 cm^{-1}). Overall, the assignments support a neutral trans and anionic cis chromophore at pH/p^2H 8.4, table 1 summarizes the assigned peaks. Local minima in the ON state ^2H_2O spectrum at 1655, 1630, 1574, 1547, 1499 and 1150 cm^{-1} are assigned to be characteristic of ν(C=O), ν(C=C), Phenol-1, ν(C=N/C=C), Phenol-3 and phenol respectively. Correspondingly, assignment of the illuminated state follows: 1680, 1651, 1616, 1562, 1514 and 1177 cm^{-1} for modes of ν(C=O), ν(C=C), Phenol-1, ν(C=N/C=C), Phenol-3 and Phenol.

Assignments for the p^2H 5.5 spectra can be made with comparison to the RsEospa p^2H 8.4 difference spectra seen in figure S6. The spectra share similar features although there are clear differences in the intensity of the carbonyl stretching region (1600-1700 cm^{-1}), and a clear decrease in intensity of the phenol-3 peak around (+)1499 cm^{-1} compared to the p^2H 8.4 spectrum. Confident assignment can be made to the dark state: 1572, 1551, 1509 and 1155 cm^{-1} for the Phenol-1, C=N/C=C, Phenol-3 and Phenol modes. The 405 nm illuminated state (figure S6d/e) assignments for this region are clear with peaks at 1611, 1565, 1516, 1176 cm^{-1} for the Phenol-1, C=N/C=C, Phenol-3 and Phenol modes. These modes are notably blue shifted compared to the p^2H 8.4 peaks. Larger shifts are seen in the carbonyl stretching region of the chromophore where peaks at 1624 and 1658 cm^{-1} are assigned to the unilluminated state for ν(C=C) and ν(C=O) vibrations. The illuminated (negative) state peak at 1645 cm^{-1} is assigned to the ν(C=C) bands. A double bleach feature similar to the peaks at 1692 and 1710 cm^{-1} (p^2H 8.4) were assigned to C=O by Lukas *et al.*[26] whilst Warren *et al.*[6] and Kaucikas *et al.*[7] do not make the same assignment based on isotope (^1H/^2H) exchange shifts which were inconsistent with harmonic frequency calculations. In RsEospa at p^2H/pH 5.5 (figure S6d/e) no ^2H_2O isotope shift is seen for the 1698 cm^{-1} positive peak whilst the negative peaks at 1690 and 1710 cm^{-1} (p^2H 8.4) shift by +2 and +3 cm^{-1} after ^1H/^2H exchange. As the isotope shift is small both the 1690 and 1710 cm^{-1} are assigned to the C=O stretch. Overall the assignment in the low pH data suggest a neutral cis conformation of the chromophore in the dark/unilluminated state (positive features). Whilst illumination with 435 nm light is assigned to isomerization to a cationic trans state, which is in particular strongly supported by the characteristic up-shifted 1713 cm^{-1} frequency and lack of ^1H/^2H sensitivity.

The peaks at 1668/1682 cm^{-1}in the ^2H_2O/^1H_2O spectra (figure S6 d/e) were tentatively assigned to the Arg 66 vasym($CN_3H_5^+$) stretch at p^2H/pH 5.5. At p^2H/pH 8.4, peaks at 1665/1682 cm^{-1} ^2H_2O/^1H_2O peaks are equivalently assigned to the Arg 66 vasym($CN_3H_5^+$) (figure S6b/c). The small shift in the ^2H_2O peak is potentially caused by a prominent side peak but gives some uncertainty to the assignment. The νsym($CN_3H_5^+$) is then assigned as 1595/1595/1597/1599 cm^{-1} in the ^2H_2O(p^2H 8.4)/^1H_2O(pH 8.4)/^2H_2O(p^2H 5.5)/^1H_2O(pH 5.5) difference spectra.

Comparison of the refined chromophore geometry of the off/on states of Dronpa crystals and low pH/neutral pH, cis/trans in rsEospa crystals (figure 4 and table S3) reveals subtle changes to coordinates which also explains the frequency shifts in carbonyl and alkene IR absorption bands in rsEospa compared to Dronpa. The shifts of the cis state spectrum can be attributed to the slight change in C_α angle and the change in the dihedral angle of the phenol ring torsion (Ψ_{cis}) from 8° to −3°/1° for Dronpa and rsEospa pH 8.4/5.5 as seen in figure 4 and table S4. The frequency shifts in trans coordinates are larger as the out of plane stretching angle (Φ_{Trans}) and Ψ_{Trans} change by around 10° compared to Dronpa. Kaucikas *et al.* show that decreased bending of the phenol (towards a planar geometry) blue shifts the C=O and C=C vibrational frequency as does decreasing the Ψ angle. Given the more planar geometry of the rsEospa crystal structure these calculations, therefore, support the blue shift of the C=O and C=C bands in the FTIR spectra of rsEospa at pD/pH 8.4 in comparison to Dronpa. The shifts at low pH, are too large to be explained by a distorted geometry and so supports protonation state assignment of a cation.

The fingerprint region differs between Dronpa and rsEospa at pD 8.4. Both share dominant peaks at 1364 cm^{-1} and around 1321 (1323) cm^{-1} and 1293 (1294) cm^{-1} but a shifted peak is seen at 1402 (1389) cm^{-1} for rsEospa (Dronpa). Similar signals are seen in the fast-switching Dronpa mutant (M159T) were Kaucikas *et al.* attribute this to skeletal deformations of the chromophore which may be associated with an equilibrium of the chromophore in a non-planar geometry [16]. The rsEospa spectra at pD 5.5 show similar changes in the fingerprint region to the pD 8.4 species which are presumably also due to slight deformations of the chromophore. However, a new negative peak at 1352 cm^{-1} is also seen which is unassigned as is the loss of the peak at 1075 cm^{-1}seen in the pD 8.4 data.

Crystallography

The pH 8.4 and 488-405 nm illumination differences support the conclusions of the occupancy refinement; it shows weaker signals than the pH 5.5 dark - 400 nm map which implies the yields of photoisomerization are lower. This is also supported by the signal location and amplitude of in the 1D difference maps (figure S7) which, are stronger in the pH 5.5 dark - 405 nm than the pH 8.4 488 nm - 488-405 nm. This increases confidence in the conclusion that the yield of cis-trans photolysis is higher in the pH 5.5 condition compared to the trans-cis at pH 8.4.

The 1D integrated electron density of the pH8.4 Dark - pH5.5 Dark map in figure S7 show strong signals in the region of residues 142-143, 146-147 and 201. These residues are associated with intra-protein crystal contacts. The strongest DED amplitude is observed on residue Pro201, which shifts in refined coordinates by 0.9 Å. This movement is then imparted onto the opposing crystal contacts at residues numbers 142-147 which justifies the strong DED at this position (figure 3c).

The 1D DED (figure S7) signals between the low and neutral pH trans states (pH 8.4 488 nm - pH 5.5 405 nm in figure 3d) are indicative of differences in cis/trans populations *and* changes to the crystal contacts which were also seen in the dark structures at low/neutral pH. Firstly, this indicates the trans occupancy between these conditions is slightly different as is supported by the occupancy refinement results in table 2, with 57 and 73 % trans population calculated for pH 5.5 405 nm and pH 8.4 488 nm respectively. Secondly, the signals around residues 142-147 indicate the trans structure shows similar crystal contact rearrangements to those seen in the cis structure although the differences are weaker, perhaps because the phases/scaling are of lower accuracy as a result of different data redundancy.

The 1-Dimensional DED between the pH 8 dark and pH 5.5 structures shows difference signals throughout the protein structure, as seen in figure S7. The pKa value for each residue in the pH 8.4 dark structure was calculated using the program PROPKA3[46] which empirically models the pKa for each titratable residue in the protein. It was proposed that structural differences could be due to changes in the protonation state of particular residues which would mean difference density would correlate with pKa values. Figure S5 shows relative pKa plotted with 1D difference density signals. It shows there is very little correlation between the calculated pKa and difference density signal which perhaps indicates that changes in pH affect solvation channels through the structure instead of causing the formation of charged residues.

Supplementary Figures and Tables

Crystallographic Tables

	pH 8.5 Dark	pH 8.5 488 nm	pH 5.4 Dark	pH 5.4 405 nm
PDB Accession Code	7TSR	7TSS	7TSU	7TSV
Merged Crystals	10790	9342	9015	13104
No. Merged Reflections	10662201 (719865)	13139114 (887337)	8063563 (544983)	11732347 (792573)
No. Unique reflection Indices	24251	24251	24251	24251
Space Group	P212121	P212121	P212121	P212121
a, b, c (Å)	39.60, 74.50, 78.98	39.52, 74.34, 78.34	39.60, 74.43, 78.32	39.50, 74.43, 79.21
Resolution Limits (Å)	78.91-1.75 (1.813-1.750)	78.91-1.75 (1.813-1.750)	78.91-1.75 (1.813-1.750)	78.91-1.75 (1.813-1.750)
Completeness (%)	100.00 (100.00)	100.00 (100.00)	100.00 (100.00)	100.00 (100.00)
$CC_{1/2}$	0.90 (0.29)	0.91 (0.52)	0.91 (0.41)	0.94 (0.31)
R_{Split} (%)	24.96 (134.96)	24.45 (80.24)	24.84 (104.79)	21.17 (128.48)
Signal to noise	3.566 (0.79)	4.035 (1.31)	3.772 (1.02)	4.045 (0.85)
Wilson b factor ($Å^2$)	24.8	23.05	24.12	26.59
CC*	0.97 (0.67)	0.98 (0.83)	0.98 (0.76)	0.98 (0.69)
Refinement				
R/R_{Free}	0.214/0.247	0.192/0.233	0.20/0.24	0.20/0.24
RMS Deviations:				
Bond lengths (Å)	0.007	0.0095	0.0092	0.0099
Bond Angles (°)	1.532	1.549	1.602	1.641

Table S1: Crystallographic statistics for rsEospa collected at room temperature using fixed target serial crystallography.

	pH 8.5 488 nm	pH 8.5 488 nm then 405 nm
Merged Crystals	11917	7696
No. Merged Reflections	18287666 (1235028)	10945978 (740850)
No. Unique reflection Indices	24251	24251
Space Group	P212121	P212121
a, b, c (Å)	39.52, 74.34, 78.98	39.57, 74.38, 78.93
Resolution Limits (Å)	78.91-1.75 (1.813-1.750)	78.91-1.75 (1.813-1.750)
Completeness (%)	100.00 (100.00)	100.00 (100.00)
$CC_{1/2}$	0.94 (0.56)	0.89 (0.51)
R_{Split} (%)	21.65 (78.19)	26.50 (83.52)
Signal to noise	4.390 (1.36)	3.837 (1.28)
Wilson b factor ($Å^2$)	23.51	22.66
CC*	0.98 (0.85)	0.97 (0.82)

Table S2: Crystallographic statistics for rsEospa collected at room temperature using fixed target serial crystallography.

	Cis Occupancy (%)	
State	R_{Free}	R
pH 8.4 Dark	69	81
pH 8.4 488 nm	46	27
pH 8.4 488 then 405 nm	48	44
pH 5.5 Dark	96	73
pH 5.5 405	63	43

Table S3: Occupancy populations of the cis chromophore refined coordinates.

	Ψ_{Trans}	Φ_{Trans}	Φ_{Cis}
Dronpa	131°	30°	8°
RsEopa pH 8.4	120°	40°	-3°
RsEopa pH 5.5	121°	42°	1°

Table S4: Out of plan bending angle, Φ and dihedral angle of the phenol ring torsion, Ψ of the crystallography chromophore coordinates of Dronpa and RsEospa as seen in figure 4.

Supplementary Figures

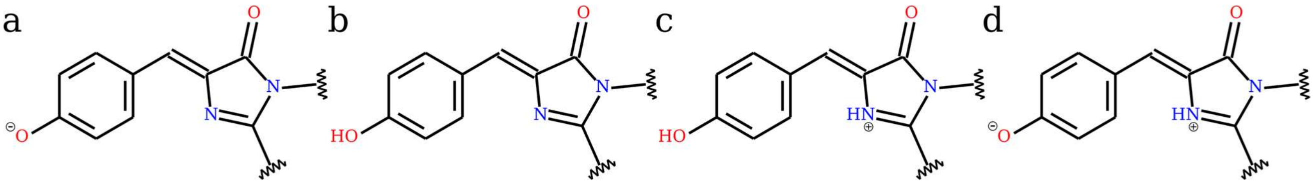

Figure S1 Protonation states of the cis chromophore: (a) neutral, (b) anionic, (c) cationic and (d) zwitterionic.

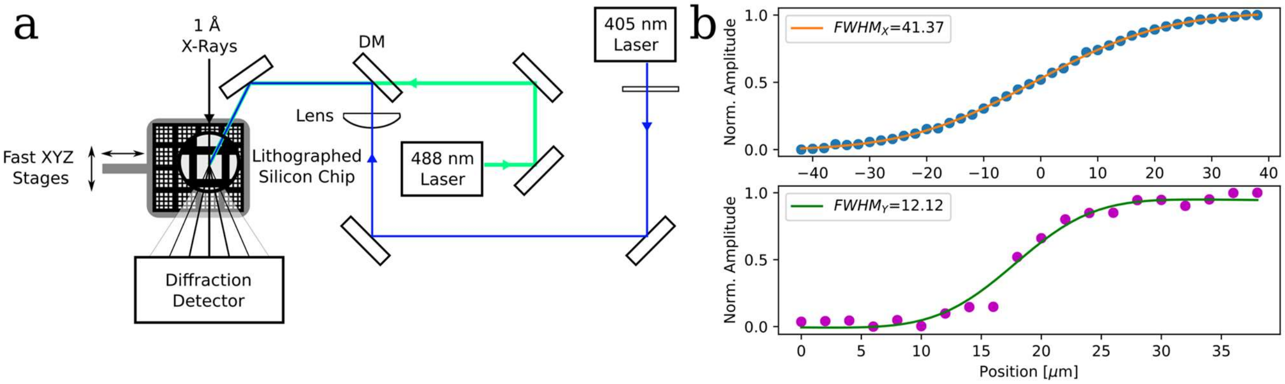

Figure S2: (a): Beamline setup. A 405 and 488 nm laser are combined collinearly on a long-pass dichroic mirror (DM). The 405 nm laser is focused using a 300 mm focal length lens onto the lithographic silicon chip. The chip is translated by 'fast' stages.

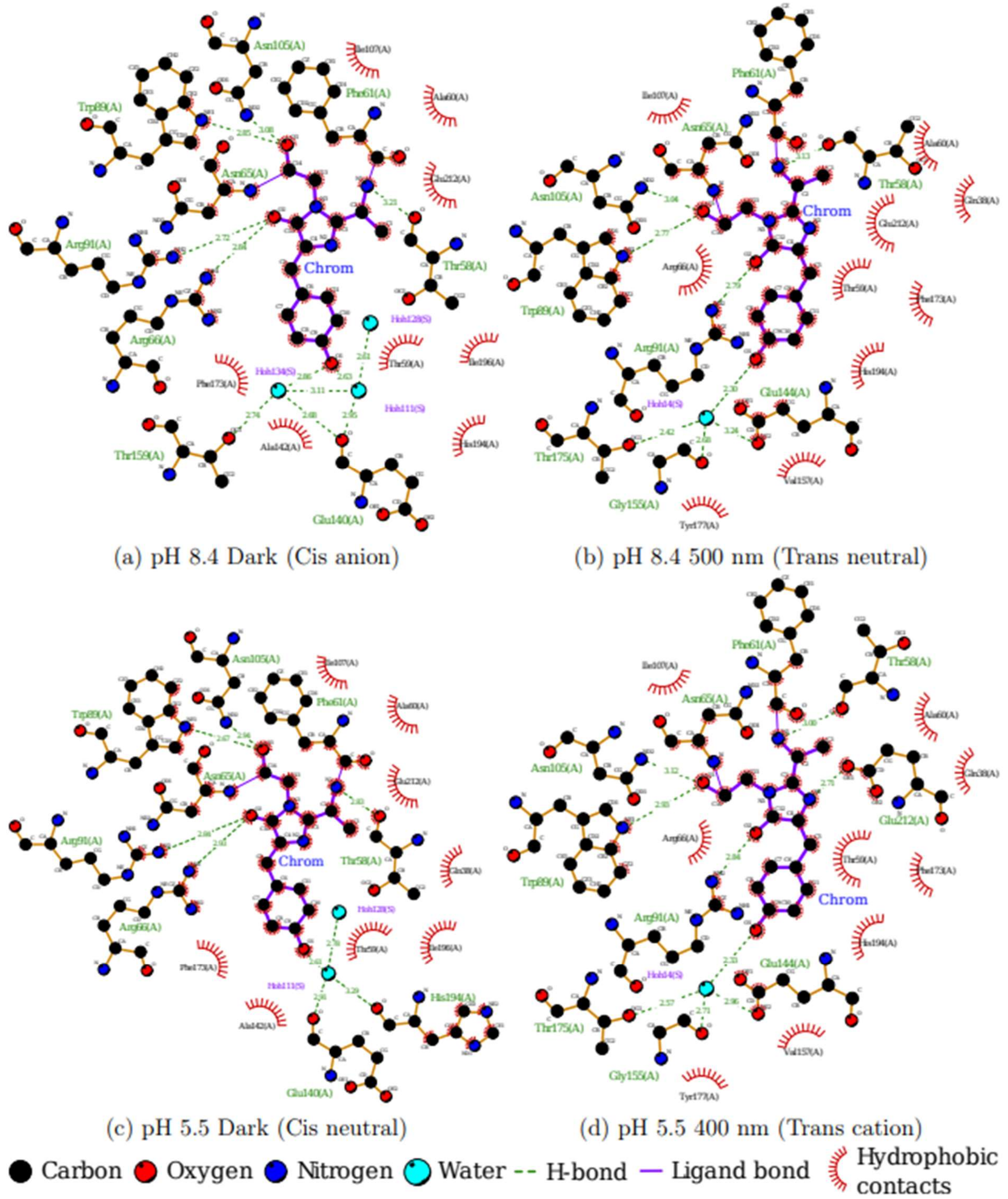

Figure S3: (a-d): LigPlot+[42] plots showing the hydrogen bonding environment (green lines) around the chromophore, interacting residues, spectator residues and distances (in Å) for different crystal structures.

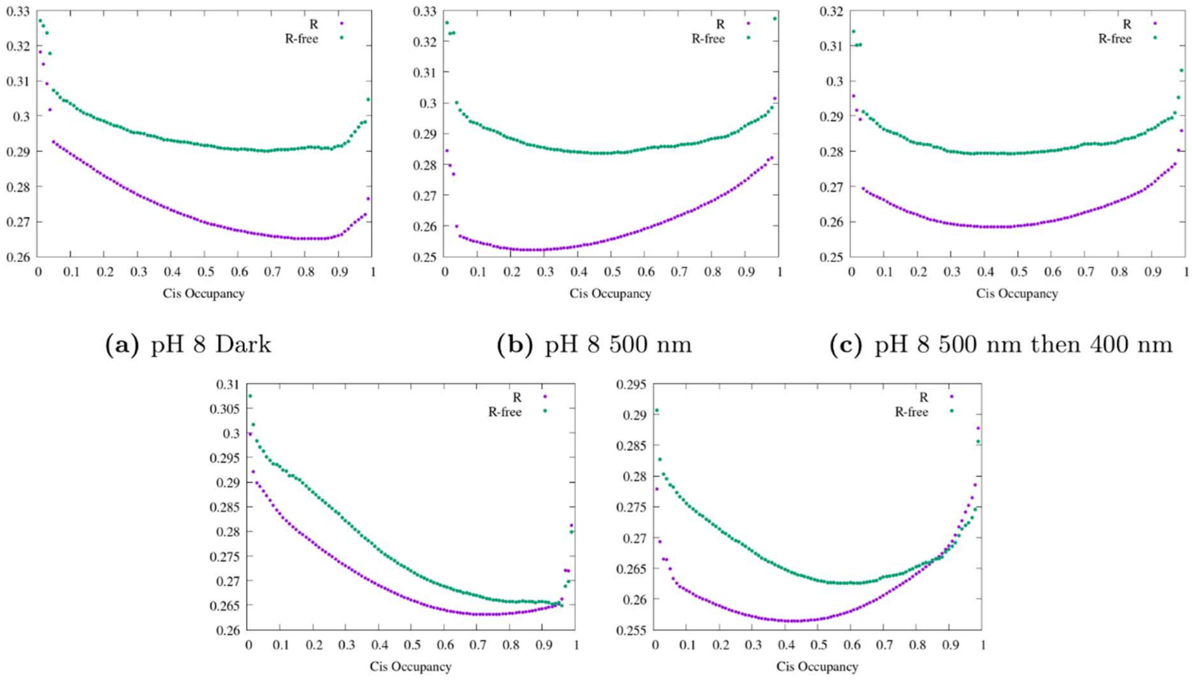

(a) pH 8 Dark **(b)** pH 8 500 nm **(c)** pH 8 500 nm then 400 nm

(d) pH5 Dark **(e)** pH5 400 nm

Figure S4: Minimization of crystallographic R-factors for rsEopa TR-SSX data at pH 5.5 and pH 8.4.

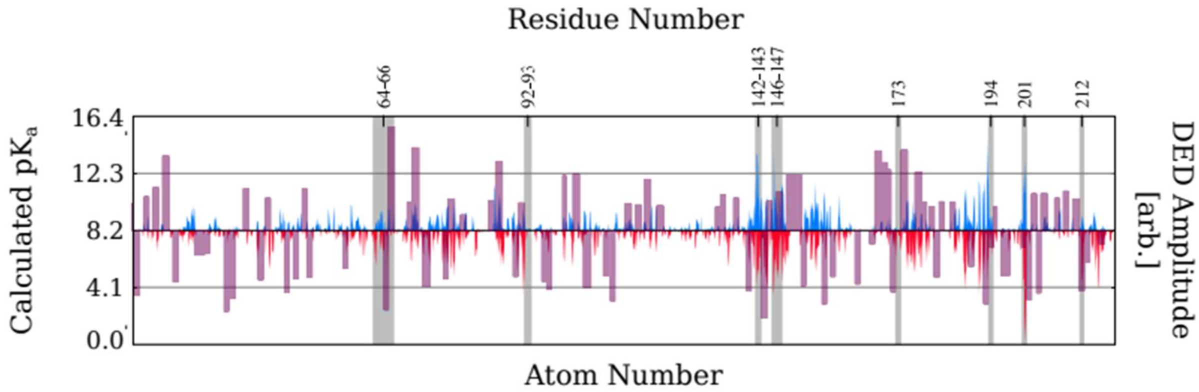

Figure S5: Calculated pKa values for titratable sites using PROPKA3 are plotted on the left axis for each residue in the protein chain (top axis label). Residues without titratable groups do not show a bar in the plot. Plotted on the right axis is the 1

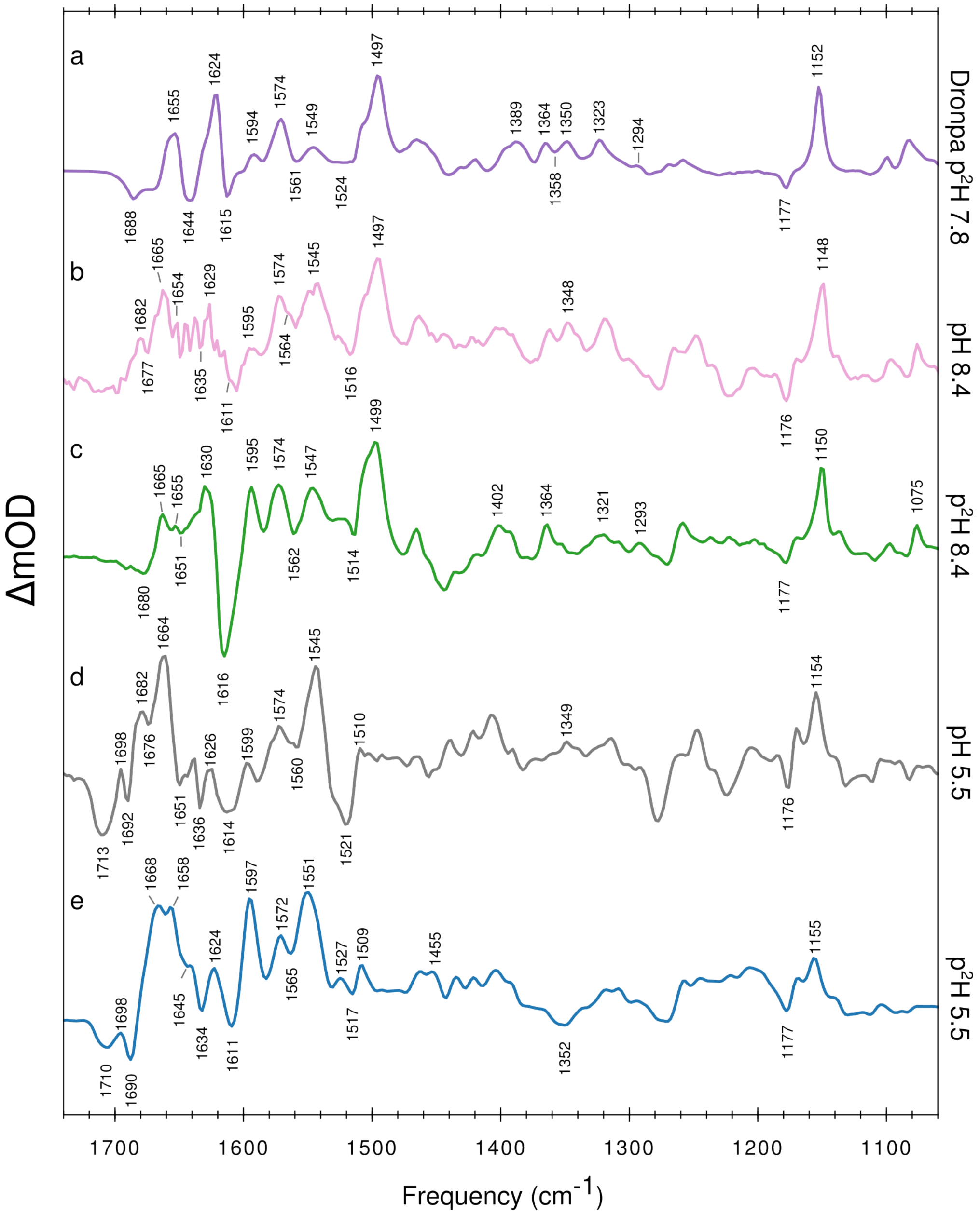

Figure S6: FTIR difference spectra for RsEospa and the wildtype Dronpa relative to the OFF state as the reactant. Spectra for RsEospa are plotted at pH/p2H 8.4. Prominent peaks discussed in the text are labeled.

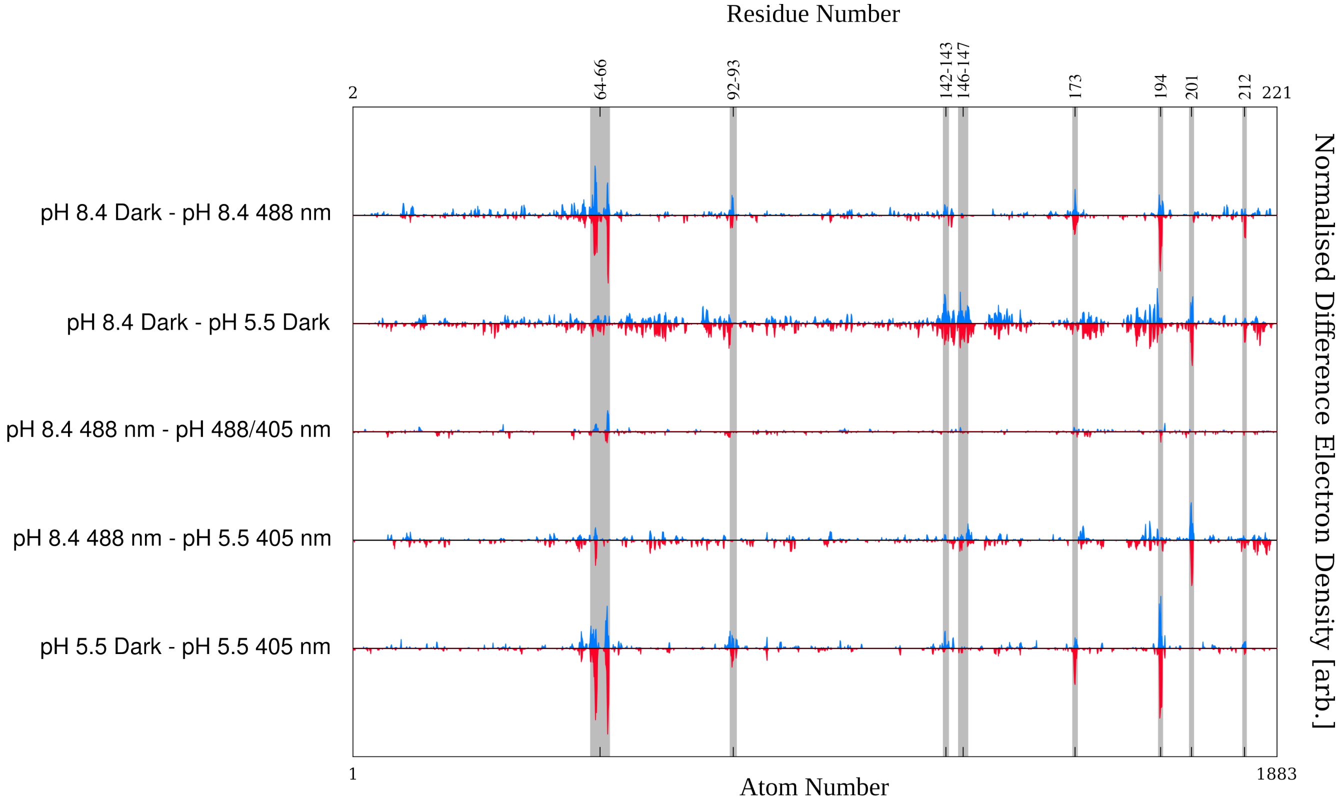

Figure S7: 1-Dimensional representation of integrated difference electron density within 2 Å at 3σ level along each atom of the protein chain, all plotted on the same scale (normalized difference electron density). Highlighted on the top axis are residue numbers of interest around the chromophore and crystal contacts as discussed in the main text. Code to produce this figure is based on the published tool by Wickstrand et al[29].

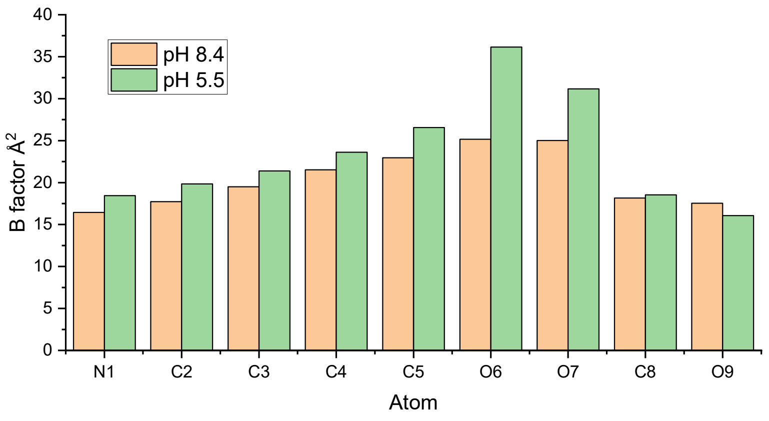

Figure S8: B-factor for residue Glu212 at pH 8.4 and pH 5.5.

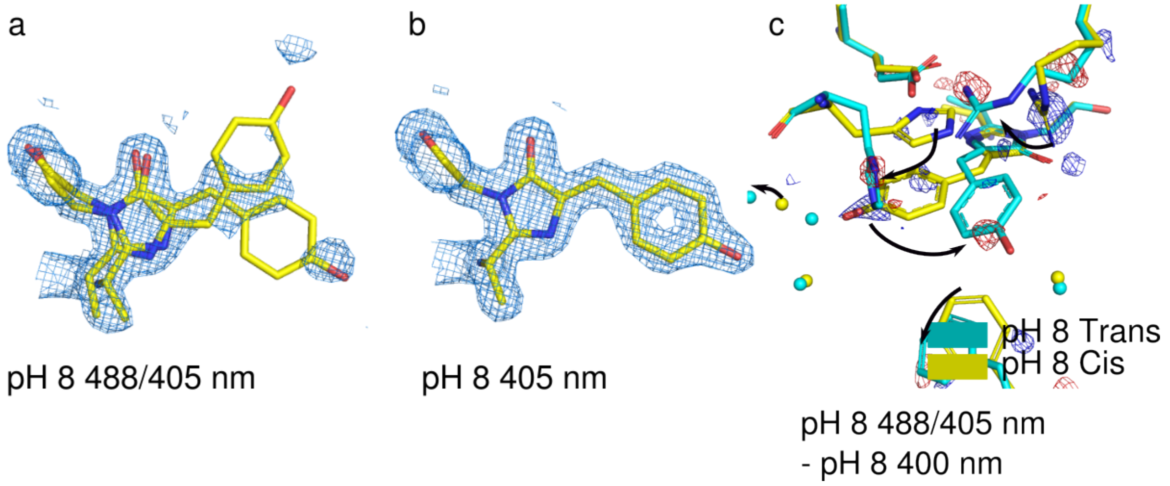

Figure S9: (a)-(b) Chromophore omitted $2F_o$-F_c maps of serial crystallography data under different illumination conditions. Positive density is shown in blue, plotted at 3σ level. (c) Q-weighted difference electron density maps of different states of rsEospa plotted in *red* and blue at 3σ level.

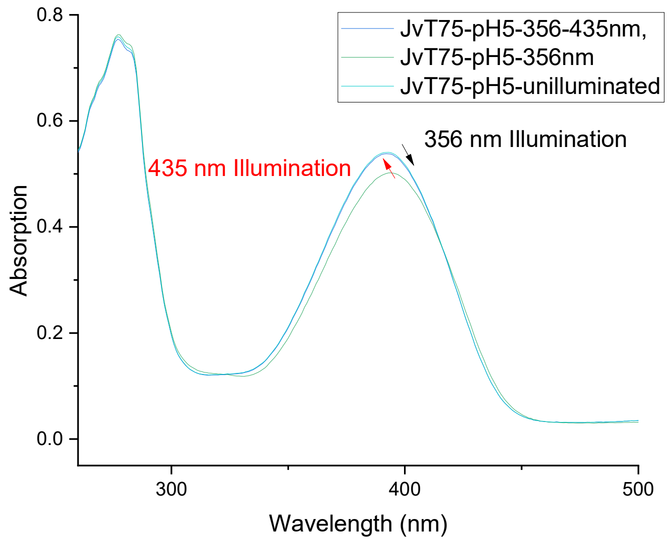

Figure S10:UV Vis spectra of rsEospa showing reconversion with illumination at 435 nm.

References

(1) Zhang, X.; Zhang, M.; Li, D.; He, W.; Peng, J.; Betzig, E.; Xu, P. Highly Photostable, Reversibly Photoswitchable Fluorescent Protein with High Contrast Ratio for Live-Cell Superresolution Microscopy. *Proc. Natl. Acad. Sci.* **2016**, *113* (37), 10364–10369. https://doi.org/10.1073/pnas.1611038113.

(2) Chang, H.; Zhang, M.; Ji, W.; Chen, J.; Zhang, Y.; Liu, B.; Lu, J.; Zhang, J.; Xu, P.; Xu, T. A Unique Series of Reversibly Switchable Fluorescent Proteins with Beneficial Properties for Various Applications. *Proc. Natl. Acad. Sci. U. S. A.* **2012**, *109* (12), 4455–4460. https://doi.org/10.1073/pnas.1113770109.

(3) Owen, R. L.; Axford, D.; Sherrell, D. A.; Kuo, A.; Ernst, O. P.; Schulz, E. C.; Miller, R. J. D.; Mueller-Werkmeister, H. M. Low-

Dose Fixed-Target Serial Synchrotron Crystallography. *Acta Crystallogr. Sect. D Struct. Biol.* **2017**, *73* (4), 373–378. https://doi.org/10.1107/S2059798317002996.

(4) Brewster, A. S.; Waterman, D. G.; Parkhurst, J. M.; Gildea, R. J.; Young, I. D.; O'Riordan, L. J.; Yano, J.; Winter, G.; Evans, G.; Sauter, N. K. Improving Signal Strength in Serial Crystallography with DIALS Geometry Refinement. *Acta Crystallogr. Sect. D Struct. Biol.* **2018**, *74* (9), 877–894. https://doi.org/10.1107/S2059798318009191.

(5) White, T. A.; Kirian, R. A.; Martin, A. V.; Aquila, A.; Nass, K.; Barty, A.; Chapman, H. N. CrystFEL: A Software Suite for Snapshot Serial Crystallography. *J. Appl. Crystallogr.* **2012**, *45* (2), 335–341. https://doi.org/10.1107/S0021889812002312.

(6) Gevorkov, Y.; Yefanov, O.; Barty, A.; White, T. A.; Mariani, V.; Brehm, W.; Tolstikova, A.; Grigat, R.-R.; Chapman, H. N. XGANDALF – Extended Gradient Descent Algorithm for Lattice Finding. *Acta Crystallogr. Sect. A Found. Adv.* **2019**, *75* (5), 694–704. https://doi.org/10.1107/S2053273319010593.

(7) Battye, T. G. G.; Kontogiannis, L.; Johnson, O.; Powell, H. R.; Leslie, A. G. W. W. IMOSFLM: A New Graphical Interface for Diffraction-Image Processing with MOSFLM. *Acta Crystallogr. Sect. D Biol. Crystallogr.* **2011**, *67* (4), 271–281. https://doi.org/10.1107/S0907444910048675.

(8) Adams, P. D.; Afonine, P. V.; Bunkóczi, G.; Chen, V. B.; Davis, I. W.; Echols, N.; Headd, J. J.; Hung, L.-W. W.; Kapral, G. J.; Grosse-Kunstleve, R. W.; McCoy, A. J.; Moriarty, N. W.; Oeffner, R.; Read, R. J.; Richardson, D. C.; Richardson, J. S.; Terwilliger, T. C.; Zwart, P. H. PHENIX : A Comprehensive Python-Based System for Macromolecular Structure Solution. *Acta Crystallogr. Sect. D Biol. Crystallogr.* **2010**, *66* (2), 213–221. https://doi.org/10.1107/S0907444909052925.

(9) Pandey, S.; Bean, R.; Sato, T.; Poudyal, I.; Bielecki, J.; Cruz Villarreal, J.; Yefanov, O.; Mariani, V.; White, T. A.; Kupitz, C.; Hunter, M.; Abdellatif, M. H.; Bajt, S.; Bondar, V.; Echelmeier, A.; Doppler, D.; Emons, M.; Frank, M.; Fromme, R.; Gevorkov, Y.; Giovanetti, G.; Jiang, M.; Kim, D.; Kim, Y.; Kirkwood, H.; Klimovskaia, A.; Knoska, J.; Koua, F. H. M.; Letrun, R.; Lisova, S.; Maia, L.; Mazalova, V.; Meza, D.; Michelat, T.; Ourmazd, A.; Palmer, G.; Ramilli, M.; Schubert, R.; Schwander, P.; Silenzi, A.; Sztuk-Dambietz, J.; Tolstikova, A.; Chapman, H. N.; Ros, A.; Barty, A.; Fromme, P.; Mancuso, A. P.; Schmidt, M. Time-Resolved Serial Femtosecond Crystallography at the European XFEL. *Nat. Methods* **2019**, *17* (1), 73–78. https://doi.org/10.1038/s41592-019-0628-z.

(10) Murshudov, G. N.; Skubák, P.; Lebedev, A. A.; Pannu, N. S.; Steiner, R. A.; Nicholls, R. A.; Winn, M. D.; Long, F.; Vagin, A. A. REFMAC5 for the Refinement of Macromolecular Crystal Structures. *Acta Crystallogr. Sect. D Biol. Crystallogr.* **2011**, *67* (4), 355–367. https://doi.org/10.1107/S0907444911001314.

(11) Emsley, P.; Lohkamp, B.; Scott, W. G.; Cowtan, K. Features and Development of Coot. *Acta Crystallogr. Sect. D Biol. Crystallogr.* **2010**, *66* (4), 486–501. https://doi.org/10.1107/S0907444910007493.

(12) Andersen, L. H.; Bluhme, H.; Boyé, S.; Jørgensen, T. J. D.; Krogh, H.; Nielsen, I. B.; Brøndsted Nielsen, S.; Svendsen, A. Experimental Studies of the Photophysics of Gas-Phase Fluorescent Protein Chromophores. *Phys. Chem. Chem. Phys.* **2004**, *6* (10), 2617–2627. https://doi.org/10.1039/B315763F.

(13) Webber, N. M.; Meech, S. R. Electronic Spectroscopy and Solvatochromism in the Chromophore of GFP and the Y66F Mutant. *Photochem. Photobiol. Sci.* **2007**, *6* (9), 976–981. https://doi.org/10.1039/b707578b.

(14) Grigorenko, B. L.; Polyakov, I. V.; Savitsky, A. P.; Nemukhin, A. V. Unusual Emitting States of the Kindling Fluorescent Protein: Appearance of the Cationic Chromophore in the GFP Family. *J. Phys. Chem. B* **2013**, *117* (24), 7228–7234. https://doi.org/10.1021/jp402149q.

(15) Barth, A.; Zscherp, C. What Vibrations Tell Us about Proteins. *Q. Rev. Biophys.* **2002**, *35* (4), 369–430. https://doi.org/10.1017/S0033583502003815.

(16) Kaucikas, M.; Tros, M.; van Thor, J. J. Photoisomerization and Proton Transfer in the Forward and Reverse Photoswitching of the Fast-Switching M159T Mutant of the Dronpa Fluorescent Protein. *J. Phys. Chem. B* **2015**, *119* (6), 2350–2362. https://doi.org/10.1021/jp506640q.
